# Des-gamma-carboxy prothrombin antagonizes the effects of Sorafenib on human hepatocellular carcinoma through activation of the Raf/MEK/ERK and PI3K/Akt/mTOR signaling pathways

**DOI:** 10.18632/oncotarget.9168

**Published:** 2016-05-04

**Authors:** Shu-Xiang Cui, Wen-Na Shi, Zhi-Yu Song, Shu-Qing Wang, Xin-Feng Yu, Zu-Hua Gao, Xian-Jun Qu

**Affiliations:** ^1^ Beijing Key Laboratory of Environmental Toxicology, Department of Toxicology and Sanitary Chemistry, School of Public Health, Capital Medical University, Beijing, China; ^2^ Department of Pharmacology, Capital Medical University School of Basic Medical Sciences, Beijing, China; ^3^ Department of Pathology, McGill University, Montreal, Quebec, Canada

**Keywords:** human hepatocellular carcinoma (HCC), Sorafenib, des-gamma-carboxy prothrombin (DCP), Ras/Raf/MEK/ERK cascade, Ras/PI3K/Akt/mTOR cascade

## Abstract

Despite significant progress, advanced hepatocellular carcinoma (HCC) remains an incurable disease, and the overall efficacy of targeted therapy by Sorafenib remains moderate. We hypothesized that DCP (des-gamma-carboxy prothrombin), a prothrombin precursor produced in HCC, might be one of the reasons linked to the low efficacy of Sorafenib. We evaluated the efficacy of Sorafenib in HLE and SK-Hep cells, both of which are known DCP-negative HCC cell lines. In the absence of DCP, Sorafenib effectively inhibited the growth of HCC and induced cancer cell apoptosis. In the presence of DCP, HCC was resistant to Sorafenib-induced inhibition and apoptosis, as determined by *in vitro* assays and in mice xenografted with HLE cells. Molecular analysis of HLE xenografted-nude mice showed that DCP activates the transduction of the Ras/Raf/MEK/ERK and Ras/PI3K/Akt/mTOR cascades. DCP might stimulate the formation of compensatory feedback loops in the intricately connected signaling pathways when kinases are targeted by Sorafenib. Our results indicate that DCP antagonizes the inhibitory effects of Sorafenib on HCC through activation of the Ras/Raf/MEK/ERK and Ras/PI3K/Akt/mTOR signaling pathways. Taken together, our findings define a DCP-mediated mechanism of inhibition of Sorafenib in HCC, which is critical for targeting therapy in advanced HCC.

## INTRODUCTION

Hepatocellular carcinoma (HCC) is one of the most lethal malignancies worldwide [1, 2]. The hallmarks of HCC are self-sufficiency, insensitivity to growth inhibitory signals, evasion of apoptosis, unlimited replicative potential, sustained angiogenesis, tissue evasion, and metastasis. Several treatment modalities are available; however, only surgical resection and liver transplantation are considered curative, if diagnosed at an early stage. Since a majority of patients are diagnosed at an advanced stage, only 15% of patients are eligible for curative treatments, and they generally have a poor prognosis with median survival times of less than 1 year [1]. Moreover, the recurrence rate is high even with a successful outcome following initial treatment. Among patients whose tumor characteristics are not appropriate for surgical therapy, systemic chemotherapy is still considered as an important strategy for improving survival. Chemotherapeutic agents include a variety of cytotoxic drugs and targeted therapeutic agents. Cytotoxic drugs have provided limited benefit for most HCC patients [2]. In contrast, targeted chemotherapy (ligands, membrane receptors and receptor-associated kinases) represents a promising strategy for the treatment of HCC. In 2007, the tyrosine kinase inhibitor (TKI) Sorafenib was approved by the Food and Drug Administration (FDA) for use in advanced HCC [3].

Sorafenib is a multi-kinase inhibitor targeting VEGFR, PDGFR, PI3K, MAPK, c-kit, and Raf, etc. [4, 5]. The efficacy of Sorafenib has been evaluated in many trials [3–8], and it is now available for the treatment of advanced HCC. Despite some progress, the overall efficacy of Sorafenib on advanced HCC remains moderate [5–9]. Several reasons for its lack of efficacy have been investigated, such as the escape from apoptosis, and formation of compensatory signaling pathways [10–12]. However, the reasons and mechanisms are still far from being understood. Previous studies have indicated that des-γ-carboxyl prothrombin (DCP) produced in HCC, is an autologous growth factor that acts to stimulate the growth of HCC, and works as a paracrine interaction factor between HCC and vascular endothelial cells to increase angiogenesis [13, 14]. DCP has also been found to stimulate HCC growth and metastasis through activation of the ERK1/2 MAPK signaling pathway [13]. Given that Sorafenib mainly inhibits HCC by targeting the tyrosine kinases ERK and MAPK, taken together with the involvement of DCP in these signaling cascades, we hypothesized that DCP might antagonize the efficacy of Sorafenib through activation of the Raf/MEK/ERK kinase cascade. More importantly, we suggest that DCP might be one of the main reasons associated with the low efficacy of Sorafenib.

## RESULTS

### DCP levels in HCC cell lines

**Figure 1:**
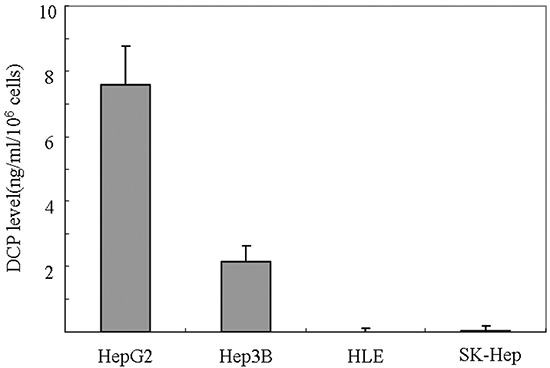
DCP expression level in HCC cell lines Cells plated at 5 × 10^6^ cells/well were incubated with media with 10% FBS for 4 h. Fresh quiescent media (8 ml/well) was applied and incubated for 48 h. The conditioned media were collected and the level of DCP produced in each cell line was determined using ECLIA. Bars indicate means ± S.D. of three independent experiments.

HCC cell lines are classified into two groups: DCP-positive and DCP-negative HCC cell lines [15, 16]. After incubation for 48 h, HepG2 and Hep3B produced DCP at rates of 7.57 and 2.14 ng/ml/10^6^ cells, respectively. HLE and SK-Hep did not produce any detectable levels of DCP (Figure 1). In this study, we used DCP-negative cell lines for their same basal DCP level [13].

### DCP attenuates Sorafenib-mediated inhibition of HCC growth in cell lines

**Figure 2:**
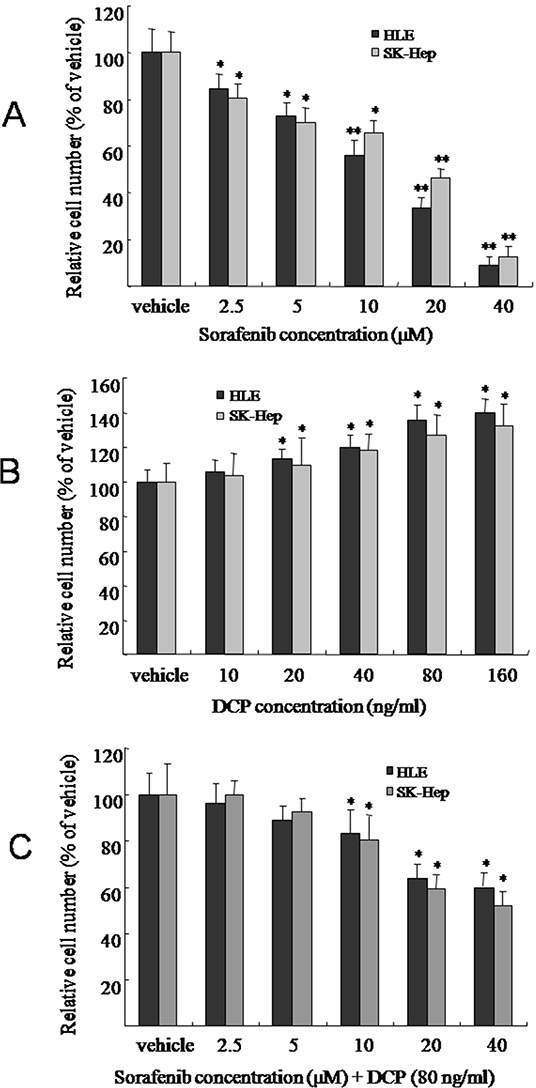
The effects of Sorafenib, DCP, and Sorafenib in the presence of DCP on HCC viability HLE or SK-Hep cells were treated with Sorafenib **A.** DCP **B.** or different concentrations of Sorafenib in the presence of DCP (80 ng/ml) **C.** for 72 h. Viable cells were evaluated by CCK-8 assay and compared with the vehicle control. The bars indicate means ± S.D. (n = 6). *p < 0.05, **p < 0.01 *vs.* the vehicle control.

To evaluate the effect of Sorafenib or DCP on HCC, the HLE or SK-Hep cell lines were exposed to Sorafenib or DCP for 72 h. Sorafenib effectively inhibited the proliferation of HCC. As shown in Figure 2A, Sorafenib treatment at concentrations of 2.5, 5, 10, 20, and 40 μM significantly decreased HCC growth in HLE cells by 15.6%, 26.8%, 40.6%, 77.6%, and 91.3%, respectively (2.5 and 5 μM, p < 0.05; 10-40 μM, p < 0.01 *vs.* the vehicle control); and it also significantly inhibited HCC growth in SK-Hep cells by 19.5%, 29.8%, 34.6%, 53.5%, and 87.4%, respectively (2.5-10 μM, p < 0.05; 20 and 40 μM, p < 0.01 *vs.* the vehicle control).

In contrast, DCP weakly increased the growth of HCC. Figure 2B illustrates the growth profiles of HLE and SK-Hep after exposure to DCP. After 72 h of treatment with DCP concentrations of 10, 20, 40, 80, and 160 ng/ml, HLE cell proliferation increased by 5.4%, 13.2%, 20.1%, 28.7%, and 35.2%, respectively (10 ng/ml, p > 0.05; 20-160 ng/ml, p < 0.05 *vs.* the vehicle control); and SK-Hep cell proliferation was increased by 3.8%, 9.8%, 18.4%, 26.7%, and 32.2%, respectively (10 ng/ml, p > 0.05; 20-160 ng/ml, p < 0.05 *vs.* the vehicle control).

In the presence of DCP, HCC cells demonstrated less sensitivity to Sorafenib, as compared to treatment with Sorafenib alone. As shown in Figure 2C, in the presence of DCP (80 ng/ml), treatment of HLE cells with Sorafenib at concentrations of 2.5, 5, 10, 20, and 40 μM decreased the growth of HCC by 3.6%, 10.8%, 18.6%, 37.6%, and 41.3%, respectively (2.5 and 5 μM, p > 0.05; 10-40 μM, p < 0.05 *vs.* the vehicle control); and HCC growth was decreased by 0, 7.5%, 9.5%, 19.5%, 40.7% and 47.7% in SK-Hep cells, respectively (2.5-5 μM, p > 0.05; 10-40 μM, p < 0.05 *vs.* the vehicle control). Statistical analysis showed that treatment with Sorafenib alone significantly decreased HCC growth in HLE cells and SK-Hep cells, while co-treatment with 80 ng/ml DCP and Sorafenib significantly decreased the efficacy of Sorafenib in these cells (Figure 2A and Figure 2C, p < 0.05).

### DCP treatment reduces the sensitivity of HCC to Sorafenib-induced apoptosis

**Figure 3:**
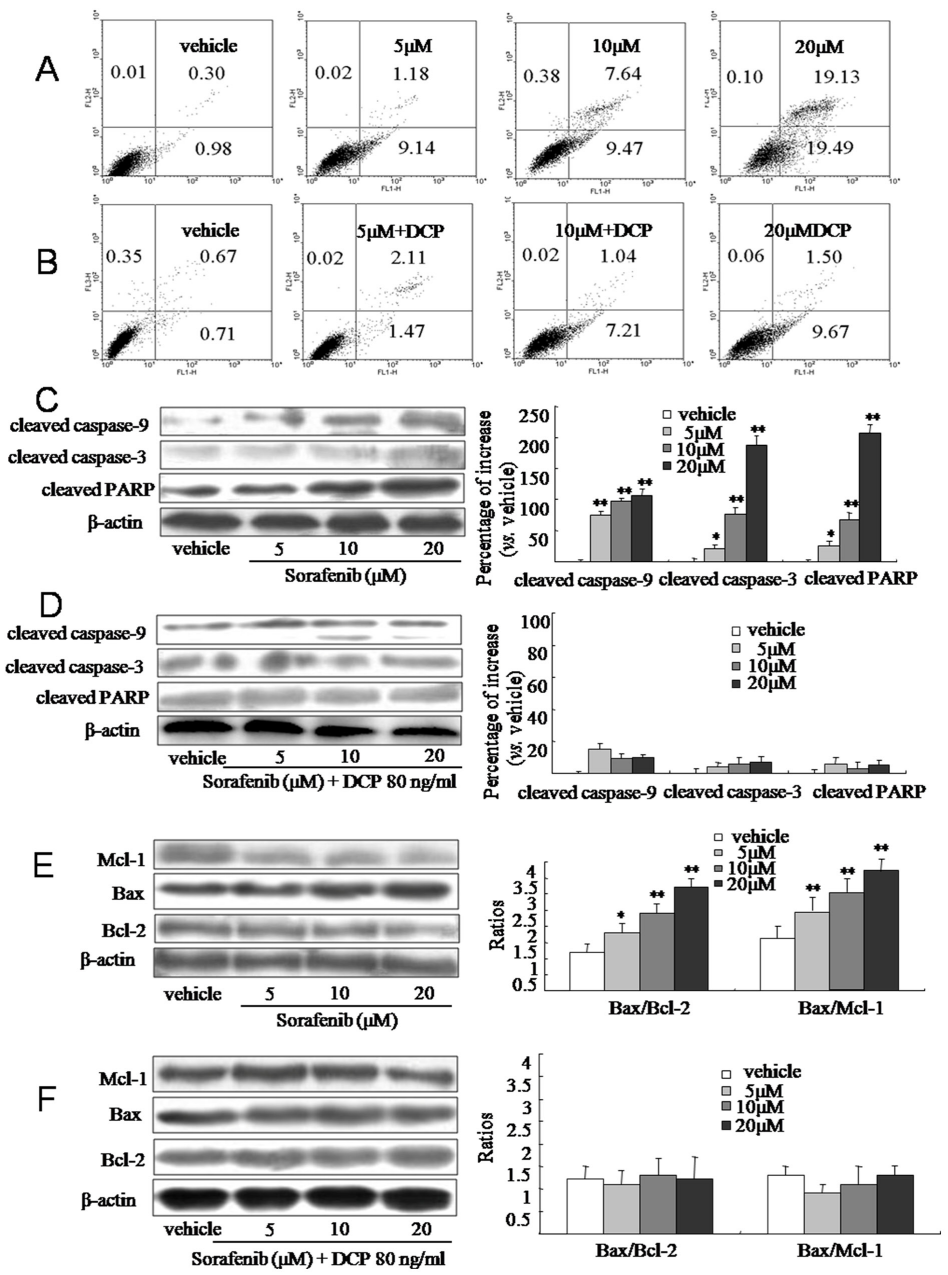
Sorafenib induces apoptosis in HCC and concurrent treatment with Sorafenib and DCP attenuates this effect **A.** and **B.** HLE cells were treated with Sorafenib or Sorafenib in the presence of DCP for 24 h. Apoptotic cells were identified using Annexin V-FITC and a PI stain assay. A: Sorafenib. B: Different concentrations of Sorafenib in the presence of DCP. **C-F.** The expression level of cleaved caspase-9, cleaved caspase-3, cleaved PARP, Bax, Bcl-2, and Mcl-1 was evaluated using Western blot analysis. Gels were run under the same experimental conditions. The cropping lines in the figure represent full-length gels. Protein levels were compared to the vehicle control, which was set to 100%. Bars indicate means ± S.D. (n = 3). *p < 0.05, **p < 0.01 *vs.* the vehicle control.

Apoptotic cells were identified in HLE cells following exposure to Sorafenib for 24 h by measuring the levels of phosphatidylserine on the cell surface. As shown in Figure 3, Sorafenib effectively induced apoptosis in HLE cells. HLE cells treated with the vehicle control had very little apoptosis (1.3%, Figure 3A); however, treatment with concentrations of 5, 10 or 20 μM Sorafenib increased the amount of apoptotic cells by 10.3%, 17.1%, and 38.6%, respectively (Figure 3A, 5 and 10 μM, p < 0.05; 20 μM, p < 0.01 *vs.* the vehicle control). In contrast, in the presence of 80 ng/ml DCP, Sorafenib treatment at concentrations of 5, 10, and 20 μM induced apoptotic cells by only 3.6%, 8.3%, and 11.2%, respectively (Figure 3B, 5 μM, p > 0.05; 10 and 20 μM, p < 0.05 *vs.* the vehicle control). Therefore, the efficacy of Sorafenib on HCC apoptosis was significantly diminished in the presence of DCP (Figure 3A and Figure 3B, p < 0.05).

The low efficacy of Sorafenib on HCC apoptosis in the presence of DCP was confirmed at the protein level. At concentrations of 5, 10 and 20 μM, Sorafenib treatment increased the levels of cleaved caspase-9 by 75.1%, 96.1%, and 106.3%, respectively (Figure 3C, p < 0.01 *vs.* the vehicle control); it increased the levels of cleaved caspase-3 by 21.3%, 76.8%, and 186.7%, respectively (5 μM, p < 0.05, 10 and 20 μM, p < 0.01 *vs.* the vehicle control), and increased cleaved PARP by 25.1%, 66.2%, and 205.7%, respectively (5 μM, p < 0.05, 10 and 20 μM, p < 0.01 *vs.* the vehicle control). However, significant increases of proapoptotic proteins were not observed when cells were concurrently exposed to Sorafenib and DCP (Figure 3D, p > 0.05 *vs.* the vehicle control). Therefore, concurrent treatment of cells with DCP and Sorafenib dramatically decreased the cleavage of proapoptotic proteins as compared to treatment with Sorafenib alone (Figure 3C and Figure 3D, p < 0.01). These results suggest that DCP has an antagonistic effect on Sorafenib treatment. Furthermore, Sorafenib significantly activated the expression of proapoptotic protein Bax, whereas it inhibited antiapoptotic proteins Mcl-1 and Bcl-2 (Figure 3E, p < 0.05 *vs.* the vehicle control). The ratios of Bax/Bcl-2 in the Sorafenib-treated cells increased from 1.2 in the vehicle control to 1.8, 2.4, and 3.2, at the concentrations of 5, 10, and 20 μM of Sorafenib, respectively (5 μM, p < 0.05; 10 and 20 μM, p < 0.01 *vs.* the vehicle control). The ratios of Bax/Mcl-1 were increased from 1.6 in the vehicle control to 1.7, 2.8, and 3.5 by exposure to 5, 10 and 20 μM of Sorafenib, respectively (5 μM, p > 0.05; 10 and 20 μM, p < 0.01 *vs.* the vehicle control). In the presence of DCP, no Sorafenib-induced changes in Bax, Mcl-1 and Bcl-2 levels were observed, as compared to the vehicle control cells (Figure 3F, p > 0.05 *vs.* the vehicle control). These results indicate that HCC cells are sensitive to Sorafenib, whereas DCP treatment reduces HCC cell sensitivity to Sorafenib.

### DCP antagonizes the efficacy of Sorafenib treatment on HLE xenografts in nude mice

**Figure 4:**
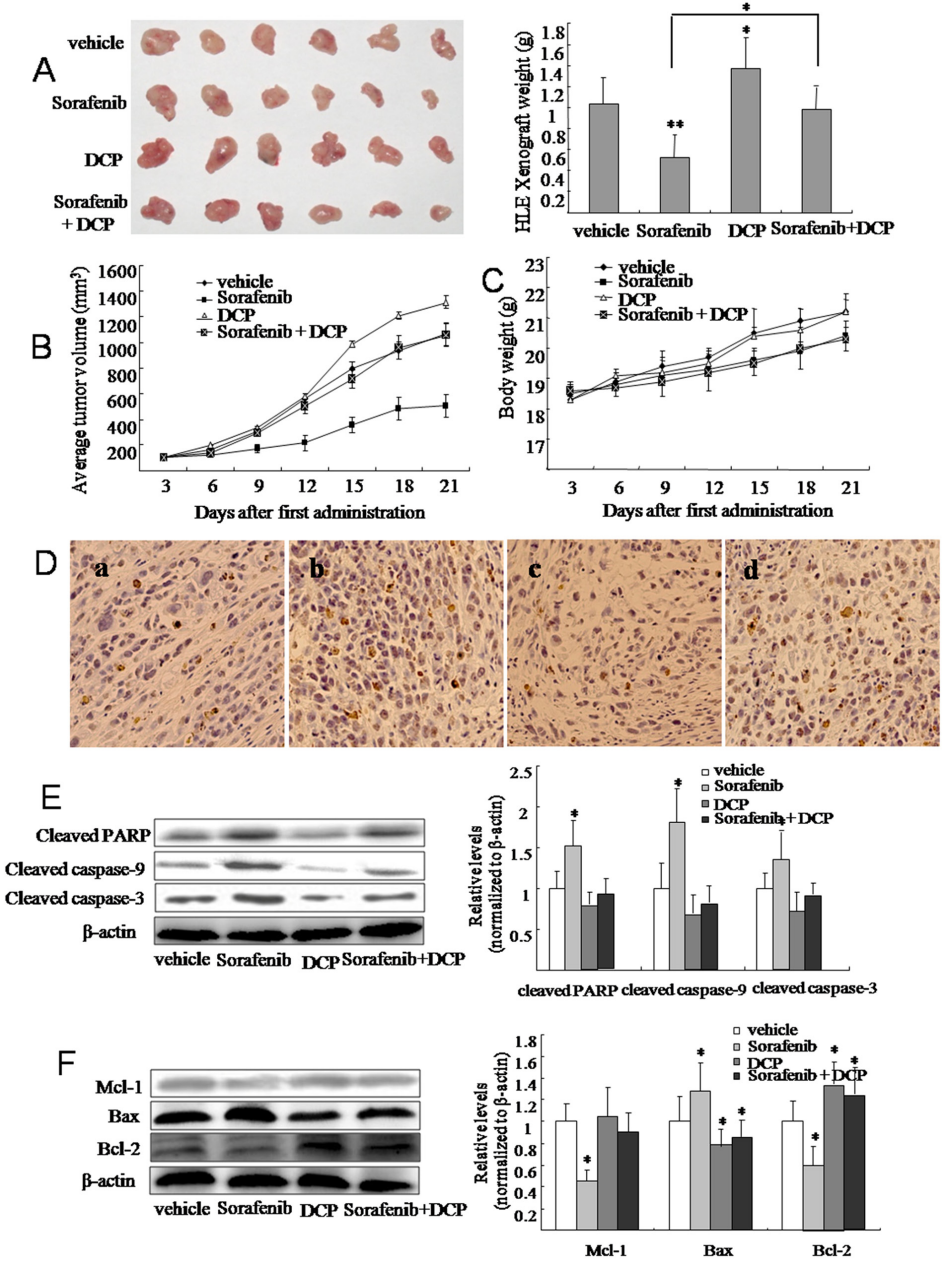
Sorafenib treatment inhibits tumor growth in HLE xenografts in nude mice and this effect is attenuated by concomitant DCP treatment Tumor tissue was inoculated into the armpit of mice with a puncture needle. When tumor volumes reached approximately 100 mm^3^, Sorafenib (25 mg/kg by gavage), DCP (40 μg/kg by intravenous injection) and Sorafenib plus DCP were administered five times per week for three consecutive weeks. **A.** After sacrifice, tumors were photographed and each tumor was weighed. Tumor volume **B.** and body weight **C.** were measured every three days. Bars represent means ± S.D. of 6 mice. **D.** TUNEL staining identified apoptotic cells in HLE xenografts. Cells with brown-stained nuclei were considered TUNEL-positive. a: the vehicle control; b: Sorafenib; c: DCP; d: Sorafenib plus DCP. **E.** and **F.** The expression level of cleaved caspase-9, cleaved caspase-3, cleaved PARP, Bax, Bcl-2, and Mcl-1 was evaluated using Western blot analysis. Gels were run under the same experimental conditions. The cropping lines in the figure represent full-length gels. Levels of proteins were compared to the vehicle control, which was set to 100%. Bars indicate means ± S.D (n = 3). *p < 0.05, **p < 0.01 *vs.* the vehicle control.

The inhibitory effect of Sorafenib on HCC was demonstrated in nude mice xenografted HLE cells. At a dose of 25 mg/kg, Sorafenib inhibited growth of the HLE xenograft by 49.3% (Figure 4A and Table 1, p < 0.01 *vs.* the vehicle control). Conversely, DCP treatment at a dose of 40 μg/kg significantly stimulated the growth of the HLE xenograft by 25.2% (p < 0.05 *vs.* the vehicle control). Concurrent dosing of Sorafenib and DCP did not significantly inhibit the HLE xenograft growth (p > 0.05 *vs.* the vehicle control). The efficacy of Sorafenib, and the effects of DCP, and concurrent use of Sorafenib with DCP on HLE xenografts were also evaluated by determining the profiles of tumor volumes during a three-week term of treatment (Figure 4B). Mice dosed with Sorafenib for three weeks had significantly smaller tumor volumes than mice dosed with the vehicle control (Figure 4B), whereas exposure to DCP resulted in a significant increase in tumor volume from day 15 (Figure 4B). Significant symptoms of toxicity were not observed in mice exposed to DCP; however, Sorafenib dosing slightly decreased the body weight of the mice (Figure 4C and Table 1, p > 0.05 *vs.* the vehicle control).

**Table 1:** DCP antagonizes Sorafenib-mediated inhibition of HCC tumor growth HLE xenograft-bearing nude mice

Groups	Number of mice (n)	Body weight (g)^a^ (initial/21 days)	Tumor weight^b^ (g, mean ± SD)	Tumor growth inhibition (%)
Vehicle	6	18.3 ± 1.1/21.2 ± 1.2	1.03 ± 0.26	-
Sorafenib 25 mg/kg	6	18.5 ± 1.4/20.5 ± 1.6	0.52 ± 0.22^**^	49.3
DCP 40 μg/kg	6	18.3 ± 1.3/21.8 ± 1.4	1.29 ± 0.29^*^	−25.2
Sorafenib + DCP	6	18.6 ± 0.9/20.4 ± 1.7	0.98 ± 0.21	4.8

Established xenografts were treated with Sorafenib by gavage, DCP by intravenous injection or Sorafenib plus DCP. All treatments were performed five times per week for three weeks in total.

a Body weight was measured as indicated over time.

b Tumors were measured after mice were sacrificed.

* p < 0.05

** p < 0.01 *vs*. the vehicle control.

The detection of apoptotic cells in HLE xenografts was performed by TUNEL staining. Vehicle-treated HLE xenografts had 5.8% apoptotic cells (Figure 4D-a), whereas Sorafenib treatment increased the apoptotic cells by 33.8% (Figure 4D-b, p < 0.05 *vs.* the vehicle control). No significant change in the number apoptotic cells was observed in the DCP-treated HLE xenografts, when compared to the vehicle-treated HLE xenografts (Figure 4D-c, p > 0.05). In the presence of DCP, there were significantly fewer Sorafenib-induced apoptotic cells in comparison to Sorafenib use alone (Figure 4D-d, p < 0.05).

Western blot analysis revealed significant changes in the expression levels of proapoptotic and antiapoptotic proteins in the HLE xenografts. Sorafenib treatment resulted in the activation of proapoptotic protein Bax, thereby leading to increases in the ratios of Bax/Bcl-2 and Bax/Mcl-1 (Figure 4F). Consequently, the expression of the cleaved proteins caspase-9, caspase-3, and PARP were significantly increased in HLE xenografts (Figure 4E). In contrast, analysis of HLE xenografts exposed to DCP demonstrated slightly decreased levels of these cleaved proteins (Figure 4E, p > 0.05 *vs*. the vehicle control), with an expected increase in Bcl-2 (Figure 4F, p < 0.05 *vs*. the vehicle control). In HLE xenografts treated with Sorafenib and DCP, the proapoptotic effect of Sorafenib was significantly lowered as compared to Sorafenib use alone (p < 0.05).

### DCP attenuates Sorafenib-mediated inhibition of Raf/MEK/ERK kinase cascade expression

**Figure 5:**
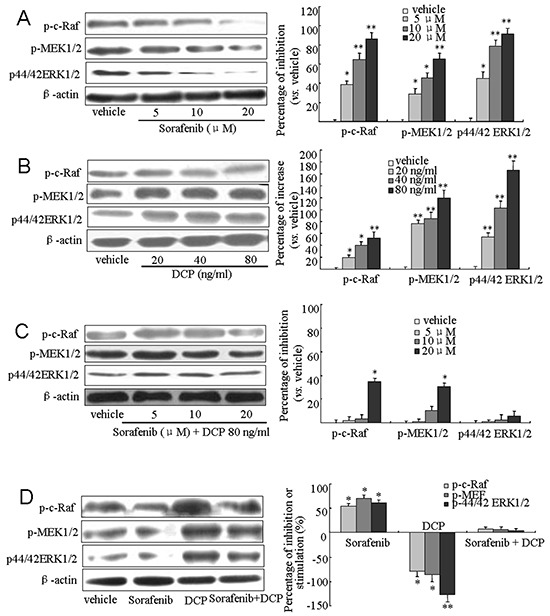
Sorafenib downregulates the Raf/MEK/ERK signaling pathway and concurrent treatment with DCP antagonizes the Sorafenib-mediated downregulation **A-C.** Western blot analysis was used to evaluate the expression of Raf, MEK and ERK kinases in HLE cells treated with Sorafenib **(A),** DCP **(B),** and Sorafenib in the presence of DCP **(C), D.** The expression levels of Raf, MEK and ERK in HLE xenografts. Gels were run under the same experimental conditions. The cropping lines in the figure represent full-length gels. Levels of proteins were compared to the vehicle control, which was set to 100%. Sorafenib inhibited the expressions of Raf, MEK and ERK kinases, whereas the levels of these kinases were stimulated by DCP (D, right panel). The bars indicate means ± S.D (n = 3). *p < 0.05, **p < 0.01 *vs.* the vehicle control.

To investigate whether DCP and Sorafenib are acting on HCC through the Raf/MEK/ERK kinases, we treated HLE cells with Sorafenib, DCP, or concomitantly with DCP and Sorafenib for 24 h and monitored the expression levels of Raf, MEK and ERK by Western blot analysis. As shown in Figure 5A, treatment with Sorafenib at 5, 10, and 20 μM significantly inhibited the expression of phospho-c-Raf^Ser259^ by 38.6%, 64.3%, and 85.9%, respectively (5 μM, p < 0.05; 10 and 20 μM, p < 0.01 *vs.* the vehicle control); it also inhibited phospho-MEK1/2^Ser217/221^ expression by 28.6%, 45.8%, and 64.9%, respectively (5 and 10 μM, p < 0.05; 20 μM, p < 0.01 *vs.* the vehicle control); and phospho-p44/42 ERK1/2^Thr-202/204^ by 44.8%, 78.7%, and 91.3%, respectively (5 μM, p < 0.05; 10 and 20 μM, p < 0.01 *vs.* the vehicle control). Conversely, treatment with DCP at 20, 40, and 80 ng/ml stimulated the expression of phospho-c-Raf^Ser259^ by 19.3%, 39.8%, and 51.9%, respectively (Figure 5B, 20 and 40 ng/ml, p < 0.05; 80 ng/ml, p < 0.01 *vs.* the vehicle control); it stimulated phospho-MEK1/2^Ser217/221^ expression by 75.9%, 84.8%, and 119.6%, respectively (p < 0.01 *vs.* the vehicle control); and it stimulated phospho-p44/42 ERK1/2^Thr-202/204^ expression by 53.9%, 102.8%, and 165.7%, respectively (p < 0.01 *vs.* the vehicle control). When HLE cells were concurrently exposed to Sorafenib and 80 ng/ml DCP, the expression levels of Raf, MEK and ERK kinases were attenuated as compared to treatment with Sorafenib alone (Figure 5C). Treatment with Sorafenib at 5, 10 and 20 μM decreased the expression level of phospho-c-Raf^Ser259^ by 2.1%, 3.2%, and 34.6%, respectively (5 and 10 μM, p > 0.05; 20 μM, p < 0.05 *vs.* the vehicle control); and it decreased the expression level of phospho-MEK1/2^Ser217/221^ by 0, 10.4%, and 30.6%, respectively (5 and 10 μM, p > 0.05; 20 μM, p < 0.05 *vs.* the vehicle control). Significant inhibition of phospho-p44/42 ERK1/2^Thr-202/204^ was not achieved even at 20 μM Sorafenib (p > 0.05 *vs.* the vehicle control). Similar changes in Raf, MEK, and ERK kinase expression levels were obtained for Sorafenib, DCP or concurrent treatment of Sorafenib and DCP in the HLE xenografts (Figure 5D). These results indicate that DCP antagonizes the effect of Sorafenib on HCC through activation of the Raf/MEK/ERK signaling pathway.

### DCP attenuates Sorafenib-mediated inhibition of PI3K/Akt/mTOR kinase cascade expression

**Figure 6:**
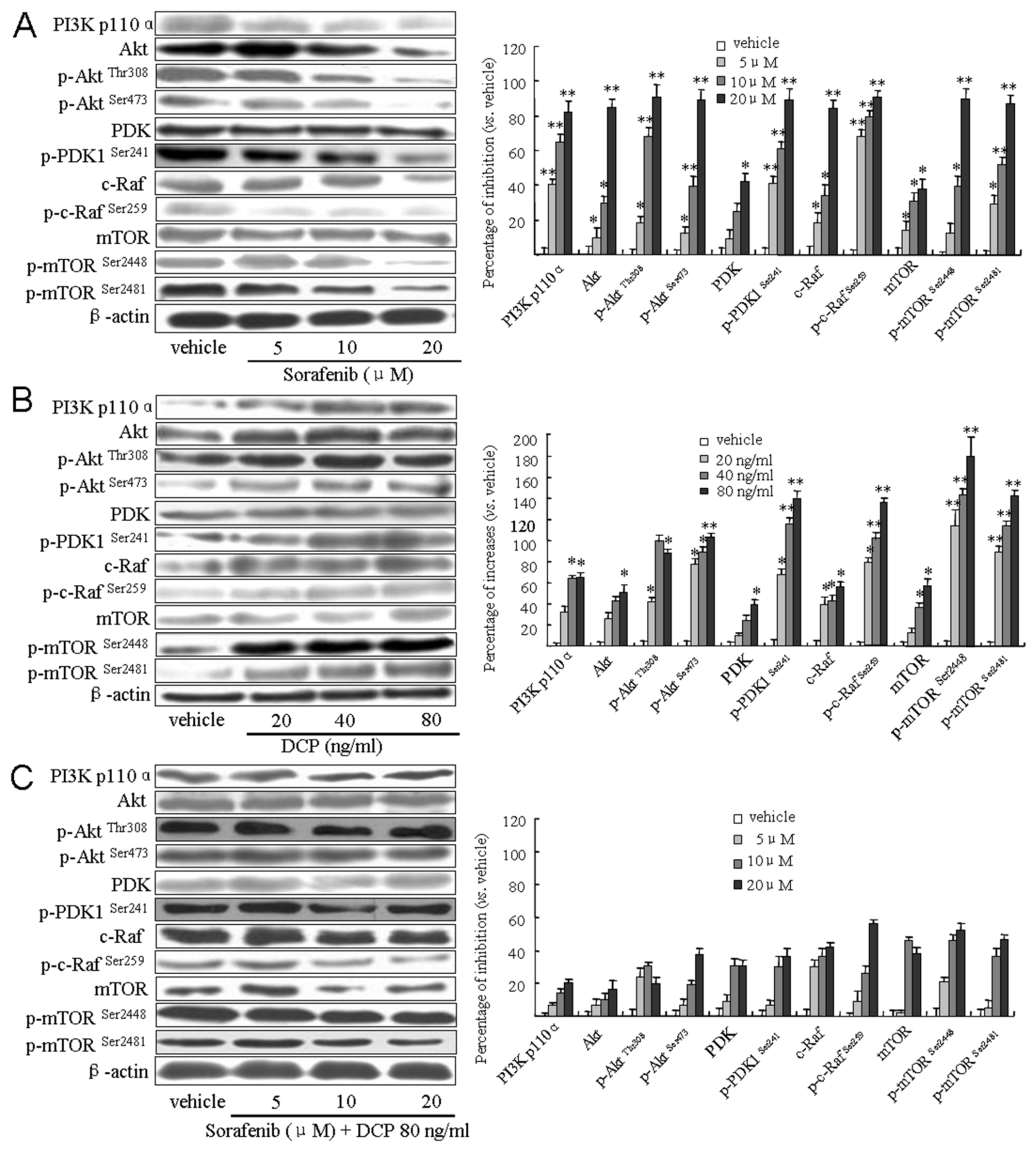
Sorafenib treatment downregulates the PI3K/Akt/TOR signaling pathway in HLE cells and concomitant treatment with DCP attenuates this effect Western blot analysis was used to evaluate the expression of PI3K p110α, Akt, PDK, c-Raf and mTOR in HLE cells exposed to Sorafenib **A.** DCP **B.** and Sorafenib in the presence of DCP **C.** Gels were run under the same experimental conditions. The cropping lines in the figure represent full-length gels. Levels of proteins were compared to the vehicle control, which was set to 100%. Bars indicate means ± S.D. (n = 3). *p < 0.05, **p < 0.01 *vs.* the vehicle control.

We investigated whether DCP antagonizes the effects of Sorafenib through activation of the PI3K/AKT/mTOR kinase cascade. As shown in Figure 6A, the expression levels of PI3 kinase P110α and Akt were significantly reduced in the Sorafenib-treated cells (PI3 P110α, p < 0.01 *vs.* the vehicle control; Akt, 5 and 10 μM, p < 0.05, 20 μM, p < 0.01 *vs.* the vehicle control). The expression levels of phosphorylated Akt at Ser473 and Thr308 were also strongly inhibited in Sorafenib treated cells (5 μM, p < 0.05; 10 and 20 μM, p < 0.01 *vs.* the vehicle control). We then examined their upstream regulators PDK and its phosphorylated form, p-PDK1 at Ser241, and the downstream effectors c-Raf and p-c-Raf at Ser259. The expression levels of these kinases were all inhibited in response to Sorafenib treatment. Sorafenib treatment of HLE cells weakly inhibited PDK expression (5 and 10 μM, p > 0.05; 20 μM, p < 0.05 *vs.* the vehicle control), whereas it strongly reduced the phosphorylated form of p-PDK1^Ser241^, as well as c-Raf and p-c-Raf^Ser259^ (p < 0.01 *vs.* the vehicle control). mTOR, a downstream effector of Akt, was also examined. Sorafenib treatment of HLE cells weakly inhibited mTOR (p < 0.05 *vs.* the vehicle control) as compared to its phosphorylated form. Sorafenib strongly reduced the expression of mTOR^Ser2448^ and mTOR^Ser2481^(Figure 6A, mTOR^Ser2448^, 5 μM, p < 0.05; 10 and 20 μM, p < 0.01; mTOR^Ser2481^, p < 0.01 *vs.* the vehicle control). In contrast, DCP activated the PI3K/Akt/mTOR cascade (Figure 6B). The levels of PI3K, Akt and its phosphorylated forms p-Akt^Thr308^ and p-Akt^Ser473^, PDK and p-PDK1^Ser241^, p-c-Raf^Ser259^ and mTOR and its phosphorylated forms p-mTOR^Ser2448^ and p-mTOR^Ser2481^ were all significantly increased in DCP-treated HLE cells (Figure 6B). Importantly, p-Akt^Ser473^ and p-Akt^Thr308^, p-PDK1^Ser241^, p-c-Raf^Ser259^, p-mTOR^Ser2448^ and p-mTOR^Ser2481^ were more sensitive to Sorafenib treatment than their non-phosphorylated forms (p < 0.01 *vs.* the vehicle controls). In the presence of DCP and Sorafenib, no significant inhibition of the levels of PI3 kinase, Akt, PDK, Raf and mTOR was detected (Figure 6C, p > 0.05 *vs.* the vehicle controls). These results indicate that DCP is involved in activation of the PI3K/Akt/mTOR signaling pathway, thereby leading to an antagonistic effect on Sorafenib inhibition of kinase expression levels.

### DCP acts through Ras/Raf/MEK/ERK and Ras/PI3K/Akt/mTOR compensatory signaling pathways

**Figure 7:**
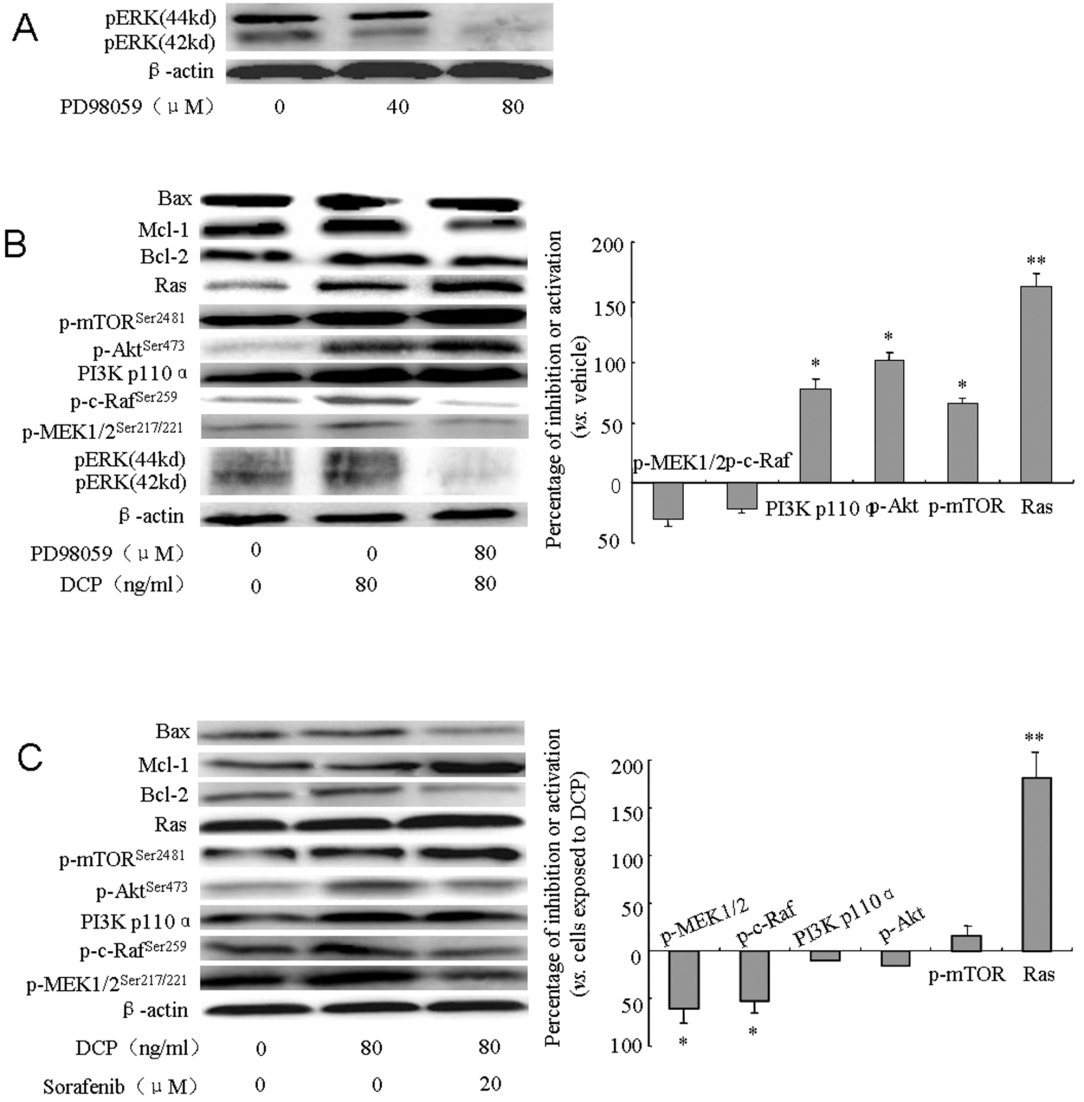
DCP acts on Ras/Raf/MEK/ERK and Ras/PI3K/Akt/mTOR compensatory signaling pathways HLE cells were pretreated with PD98059 (80 μM) for 1 h and then treated with DCP (80 ng/ml) and/or Sorafenib (20 μM) for 3 h. Western blot analysis was performed to evaluate the expression of pERK, p-MEK1/2^Ser217/221^, p-c-Raf^Ser259^, p-Akt^Ser473^, p-mTOR^Ser2481^, PI3K p110α, Ras, Bcl-2, Mcl-1, and Bax. Gels were run under the same experimental conditions. The cropping lines in the figure represent full-length gels. **A.** 80 μM PD98059 for 1 h completely inhibited the expression of p-ERK in HLE cells. **B.** The changes in the Ras/Raf/MEK/ERK cascade in HLE cells exposed to DCP when ERK was inhibited. Bars indicate means ± S.D. (n = 3). *p < 0.05, **p < 0.01 *vs.* the vehicle control. **C.** The changes in the Ras/PI3K/Akt/mTOR cascade in HLE cells exposed to DCP and Sorafenib after ERK was inhibited. The expressions of p-MEK1/2 and p-c-Raf were decreased and Ras was activated (C, right panel). Bars indicate means ± S.D. (n = 3). *p < 0.05, **p < 0.01 *vs.* the cells exposed to DCP.

To determine whether DCP is acting through the ERK pathway to antagonize the effects of Sorafenib, HLE cells were pretreated with the ERK inhibitor PD98059 (80 μM) prior to DCP (80 ng/ml), and/or Sorafenib (20 μM) treatment. ERK expression was completely inhibited by PD98059 at 80 μM (Figure 7A). Inhibition of ERK significantly changed the expression of many upstream signaling proteins and downstream effectors. PD98059 completely abolished the DCP-stimulated pERK expression while the expression level of p-MEK1/2^Ser217/221^ and p-c-Raf^Ser259^ was slightly decreased (Figure 7B, p > 0.05 *vs.* the vehicle control). When ERK was inhibited, the expression levels of Ras, PI3K p110α, p-Akt^Ser473^, and p-mTOR^Ser2481^ were consequently increased (p < 0.05 *vs.* the vehicle control; Ras: p < 0.01 *vs.* the vehicle control). However, significant changes in expression of Bcl-2, Mcl-1, and apoptosis effector Bax were not detected (Figure 7B, p > 0.05 *vs.* the vehicle control). When HLE cells were pretreated with PD98059, and then treated with DCP and Sorafenib, the expression level of p-MEK1/2^Ser217/221^ and p-c-Raf^Ser259^ were significantly inhibited (Figure 7C, p < 0.05 *vs.* cells exposed to DCP), whereas the expression level of the kinases in the PI3K/Akt/mTOR cascade showed slightly ambiguous results (Figure 7C). Sorafenib did not antagonize the DCP-induced expression of PI3K p110α, p-Akt^Ser473^ and p-mTOR^Ser2481^(p > 0.05 *vs.* cells exposed to DCP), whereas the level of Ras was strongly activated (p < 0.01 *vs.* cells exposed to DCP) when ERK was inhibited. These results indicate that the PI3K/Akt/mTOR signaling pathway was activated. Analysis of Bcl-2, Mcl-1 and Bax indicated that the ratio of Bax/Bcl-2 was significantly increased from 1.2 in the vehicle control to 1.6 (p < 0.05) and the ratio of Bax/Mcl-1 was slightly increased from 1.4 in the vehicle control to 1.6 (p > 0.05). Taken together, these results demonstrate that DCP is involved in Ras/Raf/MEK/ERK and Ras/PI3K/Akt/mTOR compensatory signaling pathways to counteract the Sorafenib-mediated inhibition of expression levels.

## DISCUSSION

DCP is an aberrant prothrombin produced by HCC due to the absence of vitamin K or to a defect in γ-glutamyl carboxylase activity [17]. However, the cause and underlying mechanism that lead to DCP production are still controversial. DCP lacks the ability to interact with other coagulation factors [13]. DCP has been used as a marker for HCC [18, 19]. High levels of serum DCP are detected in a good number of patients with tumor volumes larger than 3 cm [20]. Recently, studies have shown that high levels of DCP correlate with worse tumor behavior and poorer prognoses for patients [21]. In these studies, DCP was described as an autologous growth factor that stimulates HCC growth and works as a paracrine interaction factor between HCC and vascular endothelial cells to increase angiogenesis. DCP might be involved in the activation of many pathways, such as the Met-JAK1-STAT3, ERK1/2 MAPK and KDR-PLC-γ-MAPK, signaling pathways [13–15, 22, 23].

It is well known that HCC is less sensitive to most chemotherapeutic agents than other cancers. Furthermore, most patients are diagnosed with HCC at an advanced stage. The outcomes in these advanced HCC patients are not satisfactory. Targeted therapeutic agents represent a promising strategy for HCC treatment, and Sorafenib has been used as a first-line systemic drug for advanced HCC [3, 24]. Although significant progress has been observed, the overall efficacy of Sorafenib remains moderate. Moreover, some patients who initially respond to Sorafenib, subsequently become refractory, and after a few months of Sorafenib therapy, develop cancer progression [25, 26]. Our study tested whether DCP antagonizes the effects of Sorafenib on HCC. In fact, there were some reports describing the low efficacy of Sorafenib in the presence of DCP, for example, Kuzuya et al. found that high levels of DCP protected HCC cells from the Sorafenib-induced inhibition [27]. In this study, we investigated the underlying mechanisms involved in the low efficacy of Sorafenib on HCC in the HLE xenografted mouse model. Under these conditions, Sorafenib strongly inhibited HCC growth, whereas DCP stimulated HCC cell proliferation. These results were consistent with our previous findings that DCP stimulated HCC growth and invasion [13, 14, 28]. We then evaluated the efficacy of Sorafenib in the presence of DCP. Sorafenib achieved a moderate efficacy on HCC in the presence of DCP. We therefore conclude that DCP works antagonistically against Sorafenib.

Mounting evidence shows that the Sorafenib-induced inhibition on HCC mainly occurs from its function of inducing apoptosis [29, 30]. Sorafenib was found to induce cancer cell apoptosis through activation of the mitochondria-mediated intrinsic pathway. In essence, the externalization of phosphatidylserine is one of the earliest events in this intrinsic pathway. We thus evaluated the efficacy of Sorafenib by determining the level of phosphatidylserine. Apoptosis “effectors” caspase-9, caspase-3 and cleavage of PARP are strictly controlled by the status of antiapoptotic proteins (Bcl-2, Bcl-xl, Mcl-1) and the apoptosis “effector” Bax and Bak [31–33]. Downregulation of Mcl-1 leads to the release of cytochrome c from mitochondria into cytosol, which then leads to caspase activation, whereas overexpression of Mcl-1 protects cancer cells from apoptosis. The ratio of Bax/Mcl-1 is thus used to determine the status of cancer cells as in apoptosis or antiapoptosis [31]. In this study, DCP antagonized the Sorafenib-induced activation of Bax/Mcl-1 and Bax/Bcl-2, thereby leading to antiapoptosis. Further analysis indicated that DCP intimately links to the apoptotic pathway through activation of many kinase cascades. The PI3K/Akt/mTOR and MEK/ERK signaling pathways extensively phosphorylate apoptotic effector molecules (*e.g*., Bcl-2, Mcl-1, Bad, Bim, caspase-9 and many others) [32, 33]. Akt upregulates Bcl-2 and Mcl-1 expression via the cyclic adenosine monophosphate response element binding protein (CREB). The disposition of both pro (Bax, Bak)- and antiapoptotic (Mcl-1) proteins is regulated not just by Akt but also by ERK1/2. Phosphorylation at Ser55/65/100 (by ERK1/2) and Ser87 (by Akt) leads to degradation of Bax, Bim and Bak [33, 34]. Further, activation of PI3K drives Mcl-1 transcription through a CREB-dependent process, while mTOR regulates its translation, and the Akt/GSK3 axis controls its posttranslational proteasomal degradation [33, 35]. We thus suggest that DCP might protect cancer cells from the Sorafenib-induced apoptosis through activation of the Raf/MEK/ERK and PI3K/Akt/mTOR signaling pathways.

Our previous results indicated that DCP stimulated HCC growth and induced matrix metalloproteinase activity through activation of the Raf/MEK/ERK kinases [13]. Because Sorafenib is an inhibitor of the Raf/MEK/ERK signaling pathway, we thus established a link between the efficacy of Sorafenib and DCP by mimicking DCP in the environment of cancer growth. Sorafenib blocked activation of these kinases, thereby leading to the inhibition of cancer growth. Unfortunately, most HCC patients are determined to be DCP positive and some are diagnosed at an advanced stage with high levels of serum DCP. Therefore, these advanced HCCs may persistently grow in the environment of autologous stimulation by DCP [13].

DCP may also antagonize the Sorafenib-induced inhibition of HCC through activation of the PI3K/Akt/mTOR pathway. Numerous reports have shown that the effects of the PI3K/Akt/mTOR signaling pathway on apoptosis are mediated by the Akt phosphorylation of key apoptotic effector molecules, namely Bcl-2, Mcl-1, Bax, and caspase-9 [32, 33]. Activation of the PI3K/Akt kinases leads to antiapoptosis, whereas inhibition of the PI3K/Akt/mTOR signaling pathway induces apoptosis of cancer cells [34, 35]. Further studies have indicated that Akt phosphorylates mTOR at serine 2448 which consequently results in hyperactivation of mTOR, leading to cell proliferation and antiapoptosis. Inhibition of mTOR, on the other hand, results in cancer cell apoptosis [36]. In this study, Sorafenib inhibited the expression of Akt and mTOR, as well as other kinases, such as PDK1 and c-Raf, in the PI3K/Akt/mTOR pathway. In particular, Sorafenib demonstrated stronger activity against the phosphorylated forms of these kinases, as compared to their non-phosphorylated forms. In the presence of DCP, however, the inhibitory effect of Sorafenib on these kinases was significantly reduced, in contrast to the cells not exposed to DCP. Since DCP appears to stimulate the phosphorylated forms of the kinases in the PI3K/Akt/mTOR pathway, we therefore suggest that DCP antagonizes the effects of Sorafenib on HCC through activation of the PI3K/Akt/mTOR signaling pathway.

**Figure 8:**
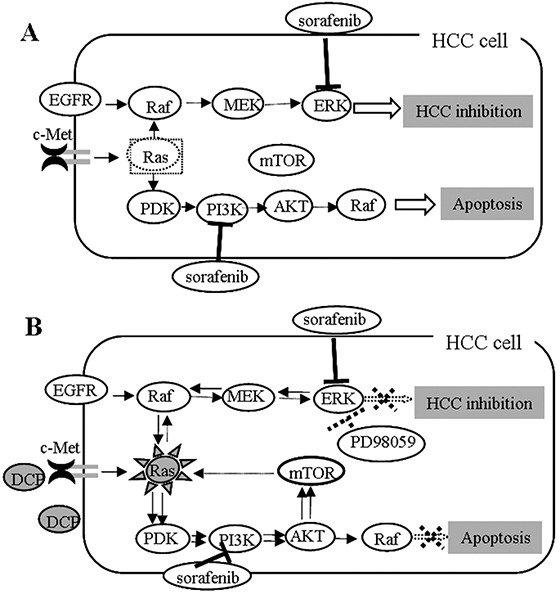
A proposed map of DCP's role in the Ras/Raf/MEK/ERK and Ras/PI3K/Akt/mTOR pathways **A.** Sorafenib inhibits HCC through targeting of multiple kinases including ERK and PI3K. **B.** Signaling pathways might interconnect with multiple points of convergence and crosstalk. When one or more kinases are inhibited, feedback loops might form through shared common inputs. DCP might antagonize the effect of Sorafenib through activation of the feedback loops.

Signaling through the Raf/MEK/ERK and PI3K/AKT/mTOR cascades is a carefully orchestrated series of events [35]. These signaling pathways are closely interconnected with multiple points of convergence, cross talk, and feedback loops through common inputs with Ras when one or more of the kinases are inhibited [37–39]. In this study, we blocked the Raf/MEK/ERK signaling pathway with PD98059 [13]. PD98059 at 80 μM completely inhibited the DCP-induced phospho-ERK1/2 and consequently prevented the antagonistic effect of DCP on Sorafenib, whereas the kinases Ras, AKT and mTOR were significantly activated. These observations indicate that compensatory pathways between the Raf/MEK/ERK and the PI3K/AKT/mTOR cascades were formed. Furthermore, PD98059 plus Sorafenib strongly inhibited the expression of pERK whereas it slightly induced Ras expression. The expression levels of kinases in the PI3K/AKT/mTOR pathway were also changed when HCC cells were exposed to PD98059. The expressions of AKT and mTOR were significantly increased with or without DCP. Sorafenib in the presence of PD98059 achieved modest inhibition on the PI3K/AKT/mTOR signaling pathway. Taken together, these observations indicate that compensatory signaling pathways were formed when ERK was inhibited. Emerging reports have shown that the PI3K/AKT/mTOR and Raf/MEK/ERK signaling pathways share common inputs and are activated by the same node Ras [33, 40–42]. For example, when mTOR is inhibited, PI3K can activate MAPK via Ras [41]. In our study, when ERK was inhibited by PD98059, Ras was activated both in the presence or absence of DCP. PD98059 could increase the Sorafenib-induced ratios of Bax/Bcl-2 and Bax/Mcl-1, but did not change the expressions of the DCP-induced antiapoptotic proteins, suggesting intricate signaling pathways. In Figure 8, we show a map of DCP involvement in the Raf/MEK/ERK and PI3K/AKT/mTOR cascade networks based on information currently available. Further study using the DCP-positive HCC cell lines HepG2 and Hep3B is underway.

In summary, our findings define a DCP-mediated mechanism of Sorafenib inhibition on HCC, which is critical for the development of a targeted HCC therapy through inhibition of the Raf/MEK/ERK and PI3K/AKT/mTOR signaling pathways.

## MATERIALS AND METHODS

### DCP and Sorafenib

Des-gamma-carboxy prothrombin (DCP) was provided by Eisai Co., Ltd., Japan. DCP was purified from the DCP-producing cell line PLC/PRF/5 in conditioned media by affinity chromatography with an anti-prothrombin antibody [15, 16]. Sorafenib was purchased from Biochempartner China (purity 99.5%). For *in vitro* assays, Sorafenib was dissolved in dimethylsulfoxide (DMSO, Sigma-Aldrich) and suspended in 5% amylum for application in mice.

### Cell lines and cell culture

HCC cell lines HLE and SK-Hep were purchased from American Type Culture Collection (ATCC). HepG2 and Hep3B were obtained from Research Resources Bank (Shanghai, China). HCC cells were maintained in RPMI-1640 supplemented with 10% (v/v) fetal bovine serum, 2 mM glutamine, and 10 mM Hepes buffer at 37°C in a humid atmosphere (5% CO_2_-95% air), and were harvested by brief incubation in 0.02% EDTA-PBS. Cell viability was estimated by using the cell counting kit-8 assay (CCK-8) (Dojindo Laboratories, Japan).

### Determination of DCP levels produced by HCC cell lines

HCC cells (5 × 10^6^ per well) seeded in 6-well plates were incubated with media with 10% FBS for 4 h. Fresh quiescent media (8 ml/well) was then applied and incubated for 48 h. The conditioned media were collected and DCP produced in each cell line was determined using an electrochemiluminescence immunoassay (Picolumi PIVKA-II TM; Eisai Co.). The electrochemiluminescence immunoassay used a mouse monoclonal anti-DCP coated on solid-phase beads and a rabbit polyclonal anti-prothrombin that has been ruthenylated. An electrochemically-triggered light reaction was quantified by an electrochemiluminescence detection system (Roche Diagnostics, Switzerland) [15, 16].

### Annexin-V and PI staining assay

HCC cells seeded in 6-well plates (2 × 10^5^ per well) were treated with Sorafenib in the presence or absence of DCP for 24 h. Cells were then harvested and washed with cold PBS. Cell surface levels of phosphatidylserine were quantitatively estimated using Annexin V-FITC and a PI apoptosis kit, according to the manufacturer's instructions (Becton Dickinson).

### HCC xenograft mice model

The effect of DCP on Sorafenib efficacy was assessed in nude mice xenografted HLE cells. We declare that all experiments were performed in accordance with the institutional guidelines outlined by the Animal Care and Use Committee at Capital Medical University. The institutional guidelines were designed by the Committee of Ethics for Animal Experimentation. The license was approved by the State Administration for Experimental Animals Committee and the permit number for this study is AEEI-2014-101. Prof. Qu's work number for animal experiments is BALAC 38760. Balb/c athymic (nu+/nu+) mice, 6 weeks of age, were purchased from the Collab Animal Center (Beijing, China). Tumors were produced by inoculating HLE cells (1 × 10^7^ per mouse) subcutaneously into the armpit of a mouse. Three weeks later, the exuberantly proliferating tumor tissue was cut into 1.5 mm thick pieces and inoculated subcutaneously into the left armpit of mice with a puncture needle. When tumor volumes reached approximately 100 mm^3^, mice were randomly divided into four groups (n = 6): vehicle control (saline by intravenous injection), Sorafenib (25 mg/kg by gavage) [43], DCP (40 μg/kg by intravenous injection) and Sorafenib plus DCP. All administrations were performed five times per week for three consecutive weeks. Mice were observed daily for any symptoms. Tumor volume and body weight were measured every 3 days. Volume was calculated using the formula, 1/2 × L × W^2^, where length (L) and width (W) were determined in mm. Tumor growth was defined as a ratio to vehicle control tumor weight. Specimens of HLE xenografts were removed for further analysis.

### TUNEL staining for apoptosis analysis

Apoptotic cells in the HLE xenografts were determined by terminal deoxynucleotidyl transferase-mediated dUTP nick end labeling (TUNEL) staining assay using the *in situ* cell death detection kit (Roche, Germany). Serial 4 μm sections were cut from formalin fixed HLE xenografts. The procedure for staining was performed according to the manufacturer's instructions. Cells with brown staining in their nuclei were considered as TUNEL positive cells. The proportion of positive cells in three mice per group was scored randomly under a microscope.

### Western blot analysis

Western blot analysis was performed to analyze the expression of kinases, antiapoptotic proteins, and apoptosis effectors in cultured cells and HLE xenografts. For *in vitro* assays, HLE cells seeded in 6-well plates (2-5 × 10^5^ per well) were treated with Sorafenib in the presence or absence of DCP for 24 h. Cells were lysed and protein was extracted as described elsewhere [15, 16]. In HLE xenografts, tumors were homogenized and protein was extracted. Western blot analysis was performed to evaluate the expression of the following proteins. The bound antibodies were visualized using an enhanced chemiluminescence reagent and quantified by densitometry using FluorChem FC3 image analyzer (Molecular Devices). Densitometric analyses of bands were adjusted with β-actin as a loading control. Primary antibodies included those to cleaved caspase-9 (9501), cleaved caspase-3 (9662), cleaved PARP (9541), Bcl-2 (2872), Bax (2772), mTOR (2983), p-mTOR^Ser2448^ (2971), p-mTOR^Ser2481^(2974), Akt (4691), p-Akt^Thr308^(2965), p-Akt^Ser473^ (4060), c-Raf (9422), p-c-Raf^Ser259^ (9421), PI3 K p110α (4255), PDK1 (3062), p-PDK1^Ser241^ (3438), p44/42 MAPK (ERK1/2)^Thr-202/204^ (9106), p-MEK1/2^Ser217/221^ (9120, Cell Signaling), PI3K p110α (sc-1331, Santa Cruz), Mcl-1 (YT2679, Immuno Way), and β-actin (ab6276, Abcam).

### PD98059 inhibitor for the Ras/Raf/MEK/ERK signaling pathway

The ERK1/2 inhibitor PD98059 was used to block the Raf-MEK-ERK kinase cascade activated in the presence of DCP, as described previously [13]. HCC cells (3 × 10^5^) seeded in 6-well plates were pretreated with PD98059 (80 μM, Cell Signaling) for 1 h, and then treated with DCP (80 ng/ml) and/or Sorafenib (20 μM) for 3 h. Western blot analysis was performed to evaluate the expression of kinases p44/42 ERK1/2^Thr-202/204^, p-MEK1/2^Ser217/221^, p-c-Raf^Ser259^, p-Akt^Ser473^, p-mTOR^Ser2481^, PI3K p110α, Ras (sc-51077, Santa Cruz), antiapoptotic proteins Bcl-2 and Mcl-1, and effector Bax. The Western blot procedure is described above.

### Statistical analysis

Data are presented as means ± S.D, and were analyzed by one-way ANOVA. Multiple between-group comparisons were performed using the S-N-K method. A p value < 0.05 was considered statistically significant. Statistical analysis was performed using SPSS/Win13.0 software (SPSS, Inc., Chicago, Illinois).
